# Efficacy analysis of oral dexamethasone in the treatment of infantile spasms and infantile spasms related Lennox–Gastaut syndrome

**DOI:** 10.1186/s12887-023-04062-6

**Published:** 2023-05-23

**Authors:** Jieling Li, Yujing Gao, Jie Cao, Fangcheng Cai, Xiuquan Zhai

**Affiliations:** 1 Department of Medical general Ward, Ministry of Education Key Laboratory of Child Development and Disorders, Chongqing Key Laboratory of Pediatrics, National Clinical Research Center for Child Health and Disorders, China International Science and Technology Cooperation base of Child development and Critical Disorders, Children’s Hospital of Chongqing Medical University, Chongqing, China; 2 Chongqing Kindcare Children’s Hospital, Chongqing, China

**Keywords:** Dexamethasone, Prednisone, Oral, Infantile spasms, IS-related Lennox–Gastaut syndrome

## Abstract

**Objective:**

Treatment with adrenocorticotropic hormone (ACTH) or a corticosteroid is the first choice for infantile spasms (IS), and vigabatrin is the first choice for children with tuberous sclerosis. Although corticosteroids may be also effective against IS and IS-related Lennox–Gastaut syndrome (LGS), the use of dexamethasone (DEX), a kind of corticosteroid, for these diseases has been rarely reported. This retrospective study aimed to evaluate the efficacy and tolerability of DEX for the treatment of IS and IS-related LGS.

**Methods:**

Patients diagnosed as having IS (including patients whose condition evolved to LGS after the failure of early treatment) in our hospital between May 2009 and June 2019 were treated with dexamethasone after failure of prednisone treatment. The oral dose of DEX was 0.15–0.3 mg/kg/d. Thereafter, the clinical efficacy, electroencephalogram (EEG) findings, and adverse effects were observed every 4–12 weeks depending on the individual patient’s response. Then, the efficacy and safety of DEX in the treatment of IS and IS-related LGS were retrospectively evaluated.

**Results:**

Among 51 patients (35 cases of IS; 16 cases of IS-related LGS), 35 cases (68.63%) were identified as responders to DEX treatment, comprising 20 cases (39.22%) and 15 cases (29.41%) with complete control and obvious control, respectively. To discuss the syndromes individually, complete control and obvious control were achieved in 14/35 and 9/35 IS cases and in 6/16 and 6/16 IS-related LGS cases, respectively. During DEX withdrawal, 11 of the 20 patients with complete control relapsed (9/14 IS; 2/6 LGS). The duration of dexamethasone treatment (including weaning) in most of the 35 responders was less than 1 year. However, 5 patients were treated with prolonged, low-dose maintenance therapy, which continued for more than 1.5 years. These 5 patients showed complete control, and 3 patients had no recurrence. Except for one child who died of recurrent asthma and epileptic status 3 months after stopping DEX, there were no serious or life-threatening adverse effects during DEX treatment.

**Conclusion:**

Oral DEX is effective and tolerable for IS and IS-related LGS. all LGS patients were evolved from IS in this study. The conclusion may not apply to patients with other etiology and courses of LGS. Even when prednisone or ACTH is failed, DEX may still be considered as a treatment option. For children who respond to DEX but do not show complete control after 6 months of treatment, prolonged treatment with low-dose DEX administered in the morning might be considered.

## Introduction

Infantile spasms (IS) and IS-related Lennox–Gastaut syndrome (LGS) are common intractable epileptic encephalopathies in children [1, 2]. Although both conditions are age-dependent, the onset age of IS predominantly occurs within the first year of life, whereas the onset of LGS occurs in children aged 1–8 years [3, 4]. Although most patients with IS are reportedly free of spasms by 3 years of age, 50–70% of patients continue to have other forms of epileptic seizures [5]. In 20–50% of patients, IS can even evolve into LGS later [6, 7].

The 2015 International League Against Epilepsy (ILAE) [8] emphasizes early diagnosis of IS and active, timely measures to control the clinical onset and resolve the hypsarrhythmia as identified using EEG. ACTH, corticosteroids, and vigabatrin are recommended as first-line drugs for IS [9], and vigabatrin is mainly used for IS caused by tuberous sclerosis (TSC). Therefore, ACTH and corticosteroids should be the preferred treatment for IS [10]. According to most studies, ACTH has a curative effect for IS in 42–87% cases, and the relapse rate is about 15–33% [11–15]. The efficacy rate of prednisone, a widely used corticosteroid, is ~ 70% [16]. Taken together, in some patients, none of the above treatments may be able to control IS, thus threatening their long-term prognosis. Early studies have suggested corticosteroids as effective against refractory LGS [17–19].

However, it is worth noting that dexamethasone (DEX), a corticosteroid, has rarely been used in the clinical treatment of IS or IS-related LGS. Although there are few reports on the use of DEX for the treatment of IS, these reports are all small sample studies [20, 21]. In our previous study, we found DEX to have significant efficacy for refractory epileptic encephalopathy with continuous spike-and-wave discharges during sleep (CSWS) [22]. To further explore the efficacy and safety of DEX in the treatment of IS and IS-related LGS, 51 patients who were diagnosed as having IS not caused by TSC and were nonresponsive to ACTH and/or prednisone were treated with DEX in the Children’s Hospital of Chongqing Medical University from May 2009 to June 2019 with the consent of parents. During the follow-up, we retrospectively analyzed the efficacy and tolerability of oral DEX for IS and IS-related LGS.

## Methods

According to the definition of ILAE [8], the inclusion criteria for patients with IS were as follows: (1) frequent clustering or repeated single-spasm seizures, (2) hypsarrhythmia or atypical hypsarrhythmia on EEG, (3) developmental retardation or regression, and (4) lack of response to standard prednisone treatment for at least 4 weeks. (5) Patients with IS caused by TSC were not included. Some of them have evolved into LGS after failing to respond to early treatment. The core characteristics of LGS are as follows: multiple types of seizures, most commonly tonic seizures; an EEG pattern consisting of slow-wave background and slow spike-and-wave discharges; and behavioral and cognitive dysfunction [4].

Upon calculating the appropriate DEX dose based on the prednisone dose, the dose of DEX was 0.15–0.3 mg/kg/d divided into two oral doses. The follow-up was scheduled every 4–8 weeks. At the same time, other anti epileptic drugs were not adjusted. If an evident response over this period was noted, then the same dose was administered again once in the morning. Finally, based on each patient’s response at follow-up, the patients were slowly weaned off DEX over several months. The clinical onset, EEG, and adverse effects were systematically evaluated during each regular follow-up. If there was any aggravation of clinical symptoms or EEG during the weaning period, an appropriately increased dose was administered and treatment was extended.

The treatment response was divided into complete control (continuous 100% reduction in seizure, accompanied by complete disappearance of hypsarrhythmia, atypical hypsarrhythmia, and slow spike-and-wave discharges), obvious control (≥ 50% decrease in seizure frequency, accompanied by significant improvement in EEG), and failure (< 50% decrease or no decrease in seizure frequency or even aggravation, accompanied by no significant improvement in EEG). The total efficiency is the sum of complete control and obvious control [23, 24].

## Results

### Clinical features

We studied 51 children (34 boys, 17 girls). The age of onset was 1–12 months [19 cases (19/51), 0–3 months; 22 cases (22/51), 3–7 months; and 10 cases (10/51), 7–12 months]. Age at time of initial DEX treatment ranged from 3 months to 7 years [18 cases (18/51), 0–1 year; 19 cases (19/51), 1–2 years; 9 cases (9/51), 2–3 years; and 5 cases (5/51), > 3 years]. When DEX treatment was started, there were 35 cases of IS and 16 cases of IS-related LGS.

Brain MRI screening was performed for all children, and 23 of them (45.10%) showed abnormal findings, such as encephalatrophy, encephalomalacia, agenesis of corpus callosum, pachygyria, heterotopic gray matter, and hydrocephalus.

All 51 children were examined by EEG (recording time ≥ 1 h, including wakefulness and sleep EEG), including 17 cases (33.33%) with typical hypsarrhythmia, 24 cases (47.06%) with atypical hypsarrhythmia, and 10 cases (19.61%) with other types of epileptic discharges (e.g., slow spike-and-wave discharges).

All patients had received standard prednisone treatment (1–2 mg/kg/d) for 4–12 weeks before DEX. In addition, 11 children had been treated with ACTH (25-50u/d) for 2–4 weeks, 5 with methylprednisolone, and 4 with vigabatrin, which were discontinued due to no significant efficacy. At the same time, all the cases were successively treated with 3–7 kinds of antiepileptic drugs (AEDs) (e.g., TPM, LEV, VPA, NP, CNP, ZNS, CLB, and LTG), 5 cases were treated with ketogenic diet (KD), 6 cases were treated with IVIG, 1 case was treated with vagus nerve stimulation, and 1 case was treated with focus cortical resection; none of these treatments led to satisfactory control.

### Efficacy and relapse

#### Treatment response of the whole group

**Fig. 1:**
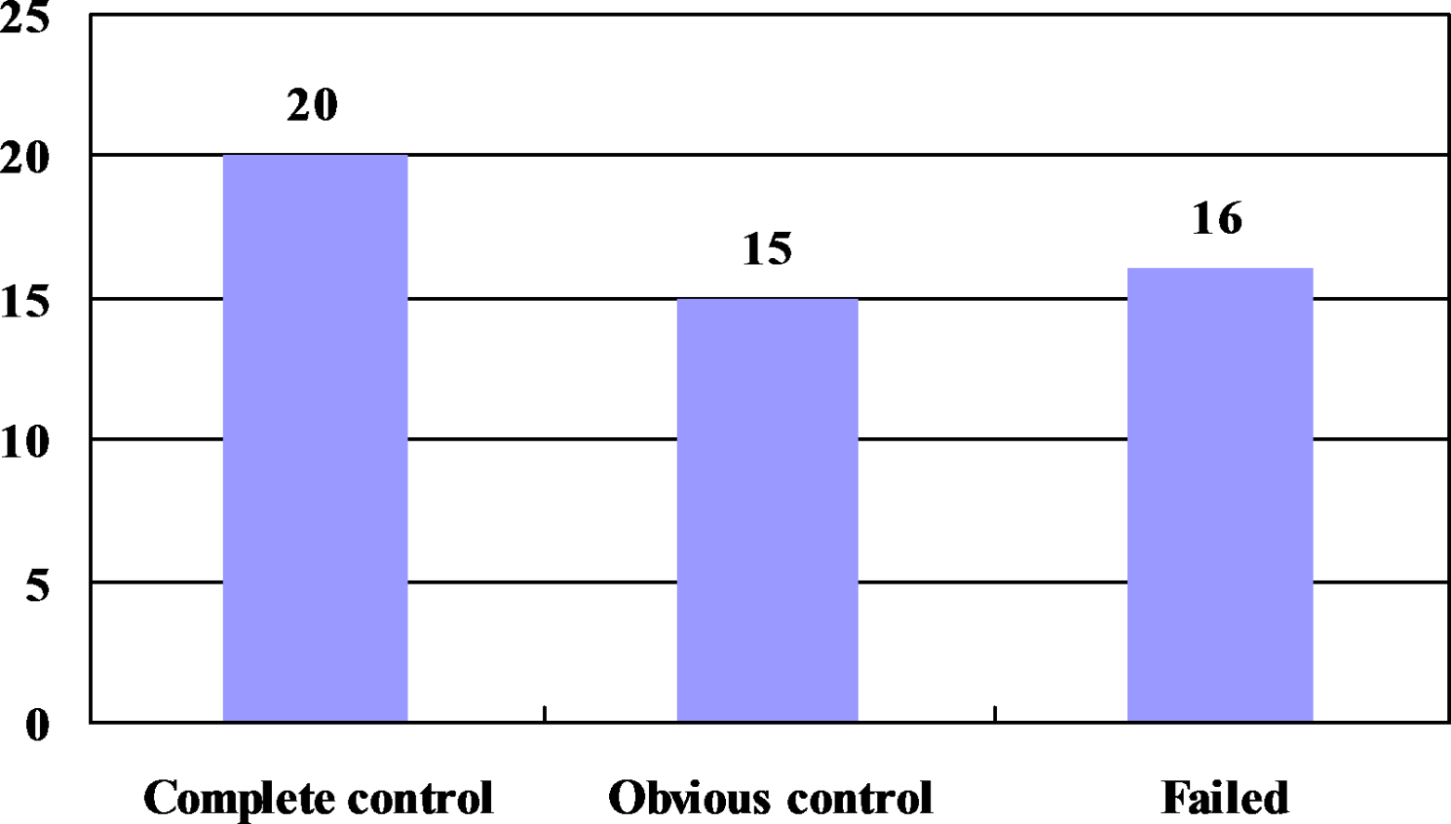
Treatment response to dexamethasone in 51 patients

Figure 1 shows the treatment response of 51 children in the whole group after oral DEX. Based on the comprehensive evaluation of clinical and EEG, 35 cases (68.63%) were identified as responders to DEX treatment, among which 20 cases (39.22%) showed complete control, 15 cases (29.41%) showed obvious control, and 16 cases (31.37%) showed treatment failure. Previously, all 35 cases showing treatment response had received standard prednisone treatment, and additionally, 6 cases were treated with ACTH and 2 cases with vigabatrin.

#### Treatment response to dexamethasone in the individual syndromes

**Fig. 2:**
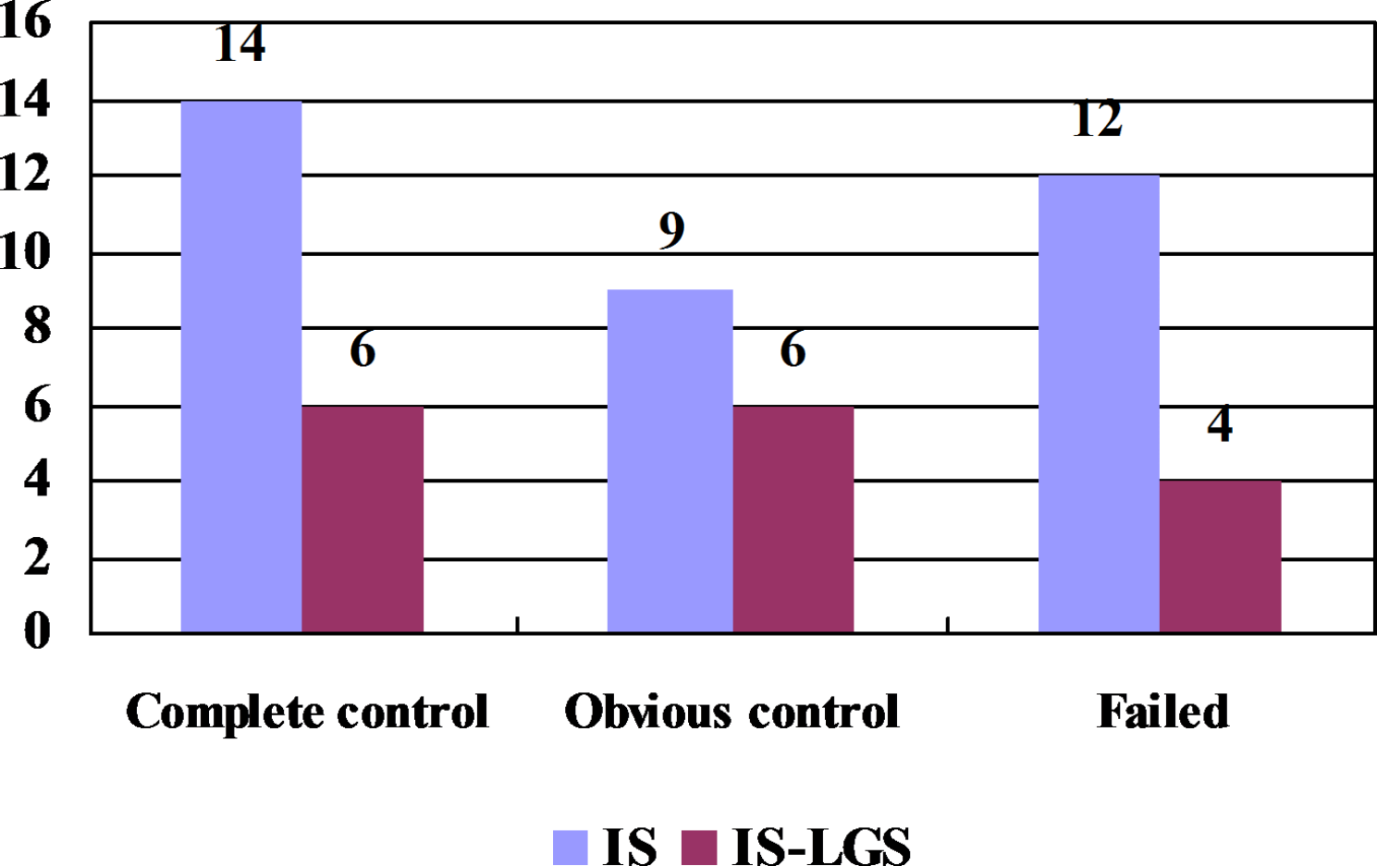
Treatment response to dexamethasone in the individual syndromes

Among the 35 patients with IS, 14 cases (40.00%) showed complete control, 9 cases (25.71%) showed obvious control, and 12 cases (34.29%) showed treatment failure. The effective response rate in IS was 65.71% (Fig. 2).

Among the 16 patients with IS-related LGS, 6 cases (37.50%) showed complete control, 6 cases (37.50%) showed obvious control, and 4 cases (25.00%) showed treatment failure. The effective response rate in IS-related LGS was 75.00% (Fig. 2).

#### Time interval to achieve complete control

During DEX treatment, the time points at which patients achieved complete control differed. For patients with IS, 14/35 patients showed complete control. Complete control was achieved within 4 weeks for 5 patients, 4–8 weeks for 3 patients, 8–12 weeks for 4 patients, and after 12 weeks for 2 patients.

**Fig. 3:**
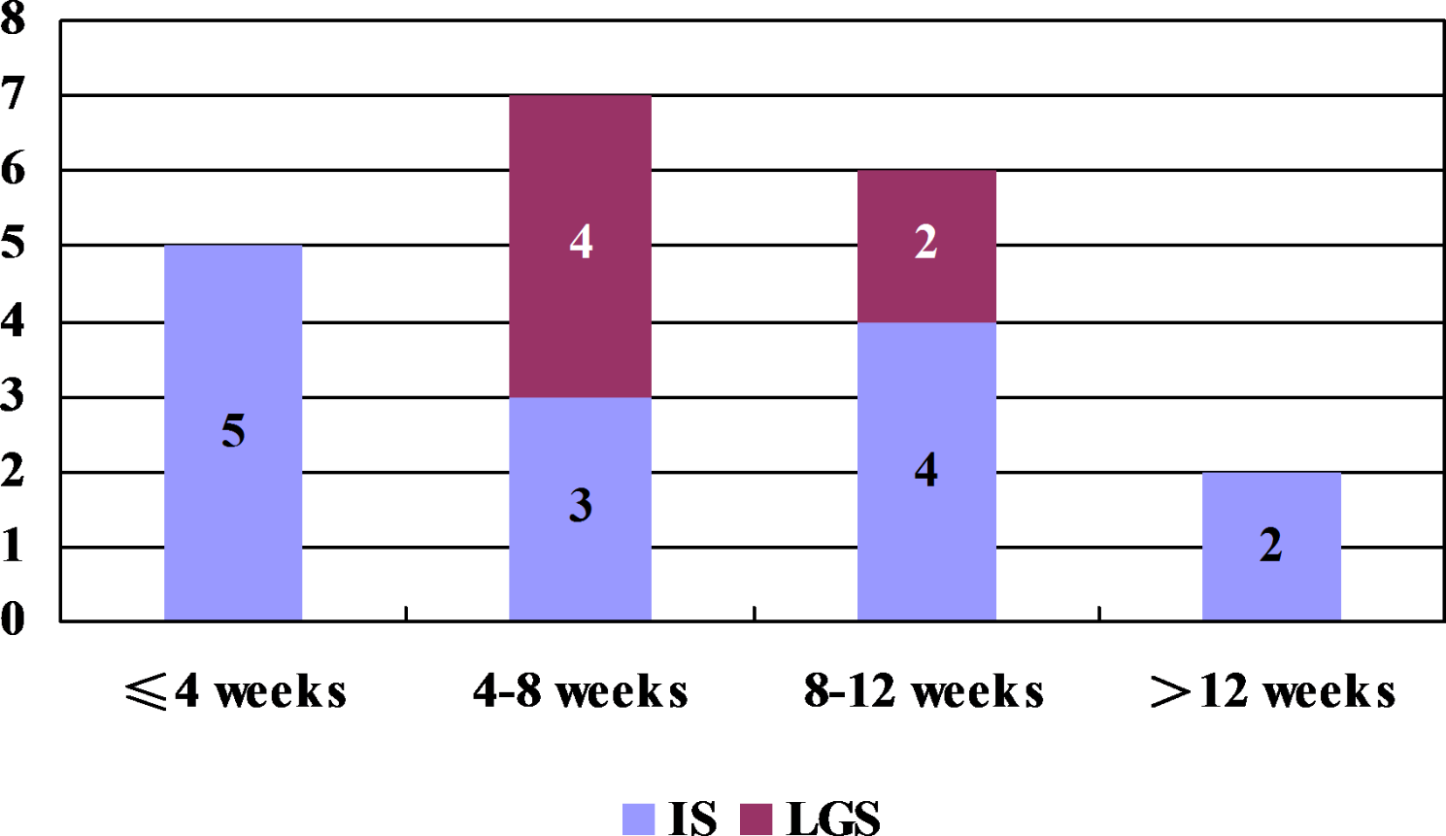
Time interval to achieve complete control

For patients with IS-related LGS, 6/16 patients achieved complete control. Complete control was achieved within 4–8 weeks for 4 patients and 8–12 weeks for 2 patients (Fig. 3).

#### Treatment periods and relapse

**Fig. 4:**
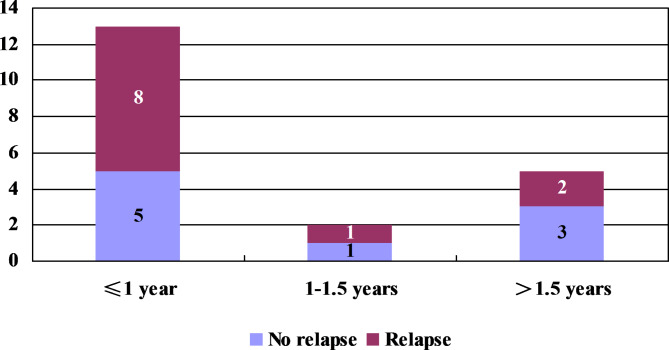
The period of treatment and relapse of complete control

The period of DEX treatment for 35 responders ranged from 3 months to 3 years and 7 months; the period of DEX treatment for 27/35 responders was within 1 year, and 20/35 achieved complete control. Of the 20 cases, 13 cases received treatment within 1 year, and 8 cases (7 cases of IS and 1 case of IS-related LGS) relapsed during the weaning and withdrawal of medication. The period of treatment was 1–1.5 years in the other 2 cases, of which 1 case of IS relapsed during weaning. In addition, 5 cases received prolonged treatment with low-dose DEX (more than 1.5 years), and 2 of these cases showed relapse during weaning and withdrawal of medication, including 1 case of IS and 1 case of IS-related LGS (Fig. 4).

Taken together, 11/20 patients who showed complete control relapsed, including 9 cases of IS and 2 cases of IS-related LGS, with a relapse rate of 55.00%. Among these 11 patients, 3 patients relapsed from not taking the medication irregularly, 2 patients harbored gene mutations (CDKL5 and KCNT1) associated with epileptic encephalopathy, and the remaining 6 patients had abnormal findings on brain MRI (e.g., encephalomalacia and pachygyria). Among the 3 patients who relapsed and were started on DEX again, 1 patient showed complete control again within 4 weeks. The other 8 patients who relapsed were successively treated with other AEDs, but only 1 patient among them showed complete control.

It is worth mentioning that all the 5 children who received prolonged treatment with low-dose DEX (> 1.5 years) achieved complete control, and 4 of them showed maintained complete control for more than 1 year. After the withdrawal of DEX, 3 cases had no recurrence, 1 case developed into LGS and again achieved complete control after resuming DEX, and the other 1 case had sound-sensitive/insensitive myoclonic nodding by accompanied atypical hypsarrhythmia and continued to have seizures despite administering DEX successively combined with ZNS, CLB, and rufinamide, among other agents.

### Tolerability

During the first follow-up of 4–8 weeks, DEX was stopped only for 1 patient (1.96%) due to severe vomiting, and 15 patients (29.41%) had mild adverse effects, including weight gain and mild Cushing syndrome. All of the 35 responders who received continuous treatment with DEX had at least one adverse effect, including Cushing syndrome (30), infection (10), increased appetite and weight (8), behavioral change (5), elevation of liver enzymes (2), hirsutism (1), and nausea (1 case). One patient died of recurrent asthma with status epilepticus 3 months after stopping DEX; none of the patients showed serious or life-threatening adverse effects during DEX treatment, and the observed adverse effects were relieved or disappeared within 6–12 months after discontinuation of DEX.

## Discussion

As epileptic encephalopathies, IS and IS-related LGS are often drug-refractory and have a poor long-term prognosis. Their cognitive and behavioral disorders caused by seizures and epileptiform discharges on EEG could be more severe than expected from the underlying cause and may gradually deteriorate over time [3, 18]. In patients with IS, frequent spasms and persistent hypsarrhythmia on EEG will cause serious damage to brain development and may eventually lead to developmental retardation or regression [25]. Existing studies show that 70–90% of patients with IS have different degrees of neuropsychiatric developmental retardation, with most of them showing moderate-to-severe developmental retardation [5]. LGS, which in some cases evolved from IS, is also a form of catastrophic epileptic encephalopathy. Although VPA, TPM, LTG, CLB, and rufinamide are proven to be safe and effective in the treatment of LGS [18, 26, 27], the seizure control and long-term prognosis of LGS are not satisfactory. In a long-term follow-up study of 89 patients [28], 91% of patients with LGS had mental retardation and 76.4% of patients had persistent seizures; notably, the clinical features and EEG discharge of 46.9% of these patients persisted into adulthood [29]. Therefore, the British and American infant spasm research centers and ILAE have put forward the concepts of “lead time” and “lag time” [8, 11, 30], emphasizing that timely diagnosis, early and effective treatment, and early control of IS clinical attack and hypsarrhythmia on EEG are critical to prevent cognitive impairment and improve the quality of life of the patients [31].

Corticosteroids are among the preferred drugs for IS [10] and are also an option for the refractory LGS [4]. In previous studies, we found that DEX had an efficacy rate of 47% in the treatment of CSWS [22]. To further explore the efficacy of DEX, we applied it to IS and IS-related LGS and found it effective in some cases. All the 51 patients were changed to oral DEX after prednisone treatment failed, and some of them were even treated with ACTH. For DEX treatment, during the follow-up, 35 cases were identified as responders (total effective rate, 68.63%), of which 20 cases (39.22%) showed complete control. This result indicates that oral DEX may be effective for some patients with IS and IS-related LGS, particularly when prednisone or ACTH therapy fails. Unfortunately, there is little research and application of DEX in this kind of disease.

Haberlandt et al. reported that the complete control rate of DEX for IS was 57%, which was equivalent to the efficacy in low-dose ACTH group [20]. In the study of Yamamoto et al. [21], DEX was given intravenously at a dose of 0.25 mg/kg each time for 12 times within 5 weeks (total dose 3.0 mg/kg). After four doses, 1 of 5 cases of IS had no nodding spasm and hypsarrhythmia and 2 cases showed > 50% decrease of epileptic seizures and EEG improvements; in the remaining 2/5 cases, the treatment was ineffective. In our study, in 35 cases of IS, the treatment was changed to oral DEX after prednisone treatment failed, and 23 cases showed obvious curative effect, with 14 cases showing complete control and 9 cases showing obvious control. The effective rate was 65.71% (23/35), and complete control was noted in 40.00% (14/35) cases. These findings suggest that oral DEX is an effective treatment for IS, and when prednisone is ineffective, DEX can be used as the first-line drug for IS.

In a study wherein DEX was used to treat LGS, Haberlandt et al. reported that using pulsatile DEX therapy could only reduce the attack frequency in 2 cases but did not achieve complete control [20]. In our study, 12/16 (effective rate, 75%) IS-related LGS patients showed evident curative effect after oral DEX treatment, with 6 cases each showing complete control (complete control rate, 37.50%) and obvious control. This result suggests that oral DEX is effective against IS-related LGS.

The major limitation of corticosteroid therapy for IS and LGS is high recurrence rate. In a long-term clinical follow-up study of IS, the recurrence rate of ACTH was 32% [32]. The recurrence rate of prednisone for IS was approximately 40% [33]. Even in the corticosteroid treatment of LGS, recurrence is very common [4, 19]. In our study, 20/35 responders achieved complete control after DEX treatment and 11 cases showed recurrence (9/14 IS; 2/6 IS-related LGS). The recurrence rate was 55% (11/20). Among the 11 patients in whom relapse occurred, 8 patients had abnormal findings on brain MRI or harbored gene mutations. The high recurrence rate could be closely related to the etiology of IS and LGS.

Corticosteroid therapy presents another challenge in the form of its adverse effects. As a long-acting corticosteroid, DEX has strong anti-inflammatory effects, and its adverse effects need to be balanced [34]. The adverse effects noted in our study were generally well tolerated. Thirty-five responders who received continuous treatment with DEX had at least one adverse effect. The most common adverse effects were Cushing syndrome (n = 30), infection (n = 10), and weight gain (n = 8). After DEX withdrawal, all adverse effects resolved as well. Except for one patient who died of recurrent asthma with status epilepticus within 3 months after stopping DEX, there were no serious or life-threatening adverse effects during the course of the treatment. Taken together, prolonged treatment with low-dose DEX administered orally was safe and tolerable under close monitoring and guidance of doctors.

Currently, there is no consensus on the optimal duration of corticosteroid therapy. As early as 1989, researchers proposed that a prolonged corticosteroid treatment for LGS could achieve “excellent” effects [17, 18]. However, thus far, studies having incorporated a prolonged corticosteroid treatment are rare. In the internationally recommended treatment therapy for IS, the period of ACTH or prednisone administration is typically 4–8 weeks [9, 10], and the response time of ACTH and prednisone is also typically 1–2 weeks [6, 11, 16]. However, we found that patients with IS-related LGS achieved complete control within 4–12 weeks (100%), and patients with IS achieved complete control within 12 weeks (85.7%); notably, 2 patients were found to achieve complete control after 12 weeks of DEX treatment. This suggests that it is feasible to appropriately extend the observation time of DEX treatment to 12 weeks. In terms of the total period of DEX treatment, the majority among 35 responders were treated within 1 year; however, in 5 cases that underwent prolonged treatment with low-dose DEX, the total treatment duration was over 1.5 years. Complete control was achieved in all of these cases, and 4/5 cases maintained complete control for more than 1 year. After DEX withdrawal, 3 cases had no recurrence, and 1 case developed into LGS, for which complete control was again achieved by resuming DEX. Existing data suggest that prolonged treatment with low-dose DEX administered orally improves the effectiveness of the therapy.

The mechanism of corticosteroid in the treatment of IS and IS-related LGS remains unclear. Araki et al. found DEX effective in reducing brain edema in children with epilepsy through subdural grid EEG monitoring in 2006 [35]. In addition, the following mechanisms are hypothesized to explain the effect of corticosteroids in the treatment of IS and LGS: immune regulation or inhibition, anti-inflammatory effect, increase of enzyme activity, regulating protein metabolism, regulating intracellular and extracellular electrolyte ratios, and regulating the intracellular glucose level [35–38]. In addition, the hypothalamic–pituitary–adrenal axis may also play an important role [39]. However, it is worth mentioning that the 35 individuals who responded to DEX treatment did not respond to an equivalent dose of prednisone in our study, suggesting that different corticosteroids have different therapeutic effect on IS and IS-related LGS and that there may be different mechanisms involved in the treatment of the diseases. Therefore, in the treatment of IS and IS-related LGS, DEX should be considered if another corticosteroid fails. However, more study is needed to analyze the different roles of corticosteroid structure and substituents in conferring their antiepileptic effect.

However, the number of cases in our study is small, and as a retrospective study, there are some limitations. In the future, studies with larger sample sizes and prospective studies are still needed to further clarify these aspects. Since we herein also compare the efficacy of dexamethasone and prednisone on IS and IS-related LGS, we hope this helps establish the effectiveness of dexamethasone in the treatment of IS and IS-related LGS.

## Conclusion

Through clinical observation, we found that oral DEX can be considered to treat IS and IS-related LGS, particularly when prednisone or ACTH has been found to be ineffective. For some patients with obvious recurrence tendencies, prolonged treatment with low-dose DEX administered orally as maintenance therapy seems to provide a better therapeutic effect. In addition, the adverse effects associated with this prolonged administration of DEX seem to be safe and tolerable. Subsequent large sample and prospective studies are still needed to further clarify.

## Data Availability

The datasets used and/or analysed during the current study available from the corresponding author on reasonable request.
